# Fractional CO_2_ Laser, Radiofrequency and Topical Estrogen for Treating Genitourinary Syndrome of Menopause: A Pilot Study Evaluating the Vulvar Vestibule

**DOI:** 10.3390/medicina60010080

**Published:** 2023-12-30

**Authors:** Madalena Leonor Pereira Campos, Ana Maria Homem Mello Bianchi-Ferraro, Carla Dias de Oliveira, Maria Cristina Caceres Nogueira, Marair Gracio Ferreira Sartori, Irene Fusco, Angela Flavia Lugollo, Neila Maria De Góis Speck

**Affiliations:** 1 Departamento de Ginecologia, Universidade Federal de São Paulo (UNIFESP), 04024-002 São Paulo, Brazil; 2 El.En. Group, 50041 Calenzano, Italy; 3 Departamento de Patologia, Universidade Federal de São Paulo (UNIFESP), 04024-002 São Paulo, Brazil

**Keywords:** genitourinary syndrome of menopause (GSM), microablative fractionated CO_2_ laser (CO_2_L), microablative fractionated radiofrequency (RF), vulvar vestibular

## Abstract

*Background and Objectives:* Genitourinary syndrome of menopause (GSM) affects more than half of postmenopausal women. This study aimed to evaluate the clinical and histological aspects of microablative fractionated CO_2_ laser (CO_2_L), microablative fractionated radiofrequency (RF) and intravaginal estrogen (ET) therapy as GSM treatments for the vulvar vestibule. *Materials and Methods:* This study included postmenopausal women with at least one moderate-to-severe complaint of GSM. Women in the CO_2_L and RF groups received three monthly sessions of outpatient vulvovaginal therapy. The procedures were performed 30 min after applying 4% lidocaine gel to the vulva and vaginal introitus. Vulvar vestibular pain was assessed after each application using a 10-point VAS. A follow-up evaluation was performed 120 days after beginning each treatment. Digital images of the vulva were obtained and a 5-point Likert scale (1 = much worse, 2 = worse, 3 = neutral, 4 = better, 5 = much better) was used to assess the global post-treatment women’s impression of improvement regarding GSM. *Results*: A significant change in clinical aspects of the vulva was observed after all treatments with a reduction in the atrophic global vulvar aspect and an enhancement of the trophic aspect. High satisfaction was also reported after treatment according to the Likert scale evaluation: CO_2_L (4.55 ± 0.97), RF (4.54 ± 0.95), CT (4 ± 1.41), *p* = 0.066. Histological evaluation revealed enhanced dermal papillae before pre-treatment, significantly reducing post-treatment in all groups (*p* = 0.002). No unintended effects were reported. *Conclusions*: CO_2_L, RF, and ET significantly improved GSM concerning the vulvar vestibule at the 4 months follow-up.

## 1. Introduction

Genitourinary syndrome of menopause (GSM) is a set of symptoms and signs resulting from clinical, anatomical, and histological changes in the lower genital and urinary tract induced by the progressive reduction in ovarian hormones [1]. GSM affects approximately 27–84% of women. It is most frequently reported in the first postmenopausal years, negatively affecting the quality of life, sexuality, and well-being [2]. GSM symptoms and signs include fissures, pruritus, dyspareunia, tropism reduction, vulvar rugae thinning, erythema and vulvar skin color [1].

In addition to clinical factors, other external factors can negatively affect sexuality [2,3]. However, women also complain about morphological changes of the vulva, such as sagging or flabby skin of the labia majora, which occur due to loss of fat, increased size of the labia minora, loss of their definition, erythema, and increased number of vulvar rugae, furrows and fissures [4].

Intravaginal estrogen therapy (ET) is the gold standard of GSM treatment [5]. However, although its efficacy and safety have been proven, some clinical conditions contraindicate its use. Moreover, the continuous use of vaginal manipulation for internal medication negatively affects treatment adherence [6].

Recently, interest in alternative treatment options has increased. Among these techniques, the use of micro ablative fractional CO_2_ laser (CO_2_L) and micro ablative fractional radiofrequency (RF) has been proposed. These techniques promote improvements in the GSM [7,8,9]. However, although there is some information in the literature regarding the histological effects of CO_2_L in the vaginal mucosa, there are no reports regarding RF or any information concerning the effects of energies in the vulvar vestibule, which is associated with relevant signs and symptoms of GSM [10,11,12].

Moreover, the use of energy-based therapies has been associated with enhanced satisfaction regarding the appearance of the genital area, being an excellent ally for women to deal with feelings of embarrassment, shame over the appearance of the genital region, anxiety about sexual intercourse and their desire to improve sexual relations [13,14].

Thus, this study aimed to evaluate the clinical and histological aspects of CO_2_L, RF and ET as GSM treatments for the vulvar vestibule.

## 2. Materials and Methods

This was a descriptive prospective pilot study conducted at Hospital São Paulo, a tertiary academic medical center of the Federal University of São Paulo (UNIFESP), between September 2019 and March 2022. This is a secondary outcome investigation from a multi-arm randomized controlled trial (LARF-Study-Arm 1) with the registration number NCT04045379.

### 2.1. Inclusion and Exclusion Criteria

This study included postmenopausal women (2–10 years since onset) with at least one moderate-to-severe complaint of GSM, defined by score 4 using a 10-point visual analogue scale (VAS) (0—“absence symptoms”, 10—“the most intense complain”) for each of the following symptoms and signs: fissures, pruritus, dyspareunia, thinning of vulvar rugae and tropism reduction. Women were considered postmenopausal if they had not experienced vaginal bleeding for at least 12 consecutive months.

Exclusion criteria were the use of hormone therapy (systemic or topical) in the last six months, current or history of gynecologic neoplasms, postmenopausal bleeding, use of immunosuppressants, medication with estrogen-like effects, uncontrolled diabetes, active genital or urinary tract infections and pelvic organ prolapse (POP-Q ≤ 2-Pelvic Organ Prolapse Quantification).

### 2.2. Evaluation

The pre- and post-treatment evaluations included observation of clinical data using VAS of GSM, gynecological examination, vulvar photography, and vestibule–vulvar biopsy. In addition, digital images of the vulva were obtained using the same camera (12 MP, resolution 4608 × 2592), without a flash, 30 cm from the genital area. All photographs were captured in the same room under the same lighting conditions, with patients in the gynecological position.

A vulvar vestibule biopsy was obtained from the right-side pre-treatment and left-side post-treatment, using a 4 mm Medina clamp. The specimens obtained were formalin-fixed immediately, and after processing and paraffin-embedding, 5 mm sections were obtained and stained with hematoxylin and eosin.

### 2.3. Allocation

Women were randomly allocated using a computer-generated block randomization table (Microsoft^®^ Excel version 16.75.2 (Microsoft Corporation, Redmond, DC, USA) to one of three study groups: CO_2_L, RF or ET; the list was kept by the nurse in charge of the protocol.

### 2.4. Treatment

Women in the CO_2_L and RF groups received three monthly sessions of outpatient vulvovaginal therapy. The procedures were performed 30 min after applying 4% lidocaine gel to the vulva and vaginal introitus. The gel was removed before application. Vulvar vestibular pain was assessed after each application using a 10-point VAS.

The equipment used for CO_2_L was a SmartXide Touch V^2^LR (MonaLisa Touch, Deka M.E.L.A., Calenzano, Italy). For vaginal applications, a 360° vaginal probe was used, and vulvar applications were performed using a vulvar probe.

The equipment used in the RF group was a Wavetronic™ 6000 Touch System with Megapulse HF FRAXX™ (Loktal Medical Electronics, São Paulo/SP, Brazil). The use of a vaginal speculum for vaginal applications performed under direct vision using a vaginal probe was necessary. A vulvar probe was used for vulval application. The pre-sets were those recommended by the manufacturers for atrophy according to the site (vagina and vulva).

Women in the ET group were instructed to vaginally use estriol cream (1 mg/g) with an applicator for 14 consecutive nights and then twice a week on alternate days for four months. Medication use was assessed by comparing the weight of the estriol tube delivered to the patient with that at each subsequent monthly visit.

Women who reported a previous episode of genital herpes received prophylaxis with Famciclovir 125 mg twice a day for three days before and two days after each application of energy.

All women were asked to abstain from sex for seven days after the energy application sessions and were instructed not to use lubricants or vaginal moisturizers during participation in the study.

### 2.5. Follow-Up

A follow-up evaluation was performed 120 days after beginning each treatment. In addition, a 5-point Likert scale (1 = much worse, 2 = worse, 3 = neutral, 4 = better, 5 = much better) was used to assess women’s global post-treatment impression of improvement regarding GSM.

### 2.6. Analysis

The vulvar pictures were evaluated by two gynecologists who were experts in vulvoscopy, blinded to the treatments and their chronological order. They scored the images from 0 (worst aspect) to 5 (best aspect) based on tropism and the number of vulvar skin folds, fissures, erythema and color. Moreover, according to the same criteria, the assistant physician evaluated the vulvar vestibule during the gynecological examination as “trophic”, “hypotrophic” or “atrophic”.

Two blinded expert pathologists performed histological analysis. The histomorphological evaluation involved sample suitability. This was defined as any tissue without any artifactual features such as decreased fixation quality or clamping artefacts and with proper representation of the epithelium and stroma.

The parameters for histological evaluation were as follows: corneous layer of skin thickness (hypertrophy was considered hyperkeratosis), epithelial thickness (measured with a Breslow ruler, considering the thickest part of the tissue upon 10× optical enhancement), number of epithelial cell layers (5 to 10 was considered reduced, 11 to 20 was considered normal), epithelial cell maturation status (presence of basal, intermediate and superficial cells was considered normal; reduction in superficial and intermediated cells was considered maturation reduction) and the presence of dermal papillae (invagination of the epithelial tissue within the stroma, with more than one-third of its thickness with the normal lining of the corresponding basal membrane.

The presence of at least five papillae in at least two distinct areas was considered normal. Identification of more than 10 papillae per area was considered enhanced, whereas the finding of less than two papillae was considered reduced). Vascularization (i.e., the assessment of the number of capillaries among two distinct stromal areas) was assessed per the following criteria: reduced, less than 2 capillaries, normal 2 to 5 capillaries, enhanced more than 10 capillaries. Capillary distribution was also assessed, considering the normal pattern of isolated subepithelial and occasional vessels in the chorion/submucosa. According to a previous definition, histological atrophy was considered a significant decrease in the number of epithelial layers, more than half the thickness of the epithelium represented by basal cells, a decrease in or absence of the intermediate and superficial layers of the squamous epithelium, a reduction in dermal papillae and a reduction in vascularization [14].

### 2.7. Statistical Analysis

Statistical analyses were performed using IBM-SPSS version 22.0 and (Microsoft^®^ Excel version 16.75.2 (Microsoft Corporation, USA). Quantitative characteristics were compared between the groups using Student’s *t*-test or the Mann–Whitney test. Qualitative characteristics were described using absolute and relative frequencies and checked for association using chi-square or likelihood ratio tests.

Quantitative characteristics were grouped and compared using analysis of variance (ANOVA) or Kruskal–Wallis tests. Qualitative features were described according to groups and assessment time and compared using generalized estimating equations (GEE) with a normal distribution. Statistical significance was set at *p* < 0.05. Because this was a pilot study on the histological and clinical aspects of the vulvar vestibule among women with GSM, the study’s power was not previously assessed. During the enrollment phase of this study, the estimated minimum sample size was 10 patients in each group.

## 3. Results

**Figure 1:**
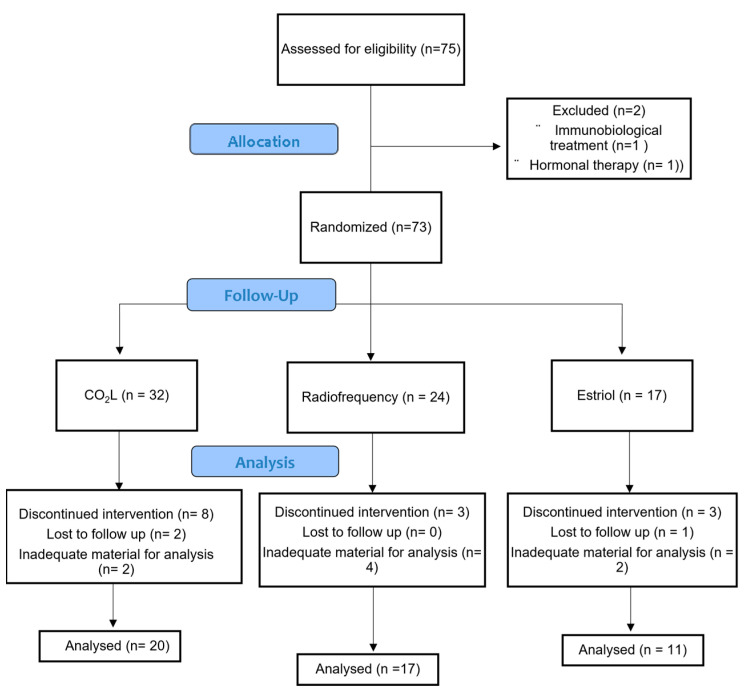
Consort flow diagram.

Seventy-three women were eligible and randomized as follows: 32 in the CO_2_L, 24 in the RF and 17 in the ET groups. Forty-eight women completed the treatment follow-up and had appropriate pre- and post-treatment material for histological analysis, 20 in the CO_2_L group, 17 in the RF group and 11 in the ET group (Figure 1).

The clinical and demographic pre-treatment parameters are listed in Table 1. The participants’ mean age was 54.6 (±4.8) years, and the mean time since menopause was 4.9 (±3). The women had a mean body mass index (BMI) of 28.5 (±4) kg/m^2^, a mean parity of 2 (±1.1), were predominantly white (65%) and were sexually active (95%). All parameters were similar between the groups (Table 1).

**Figure 2:**
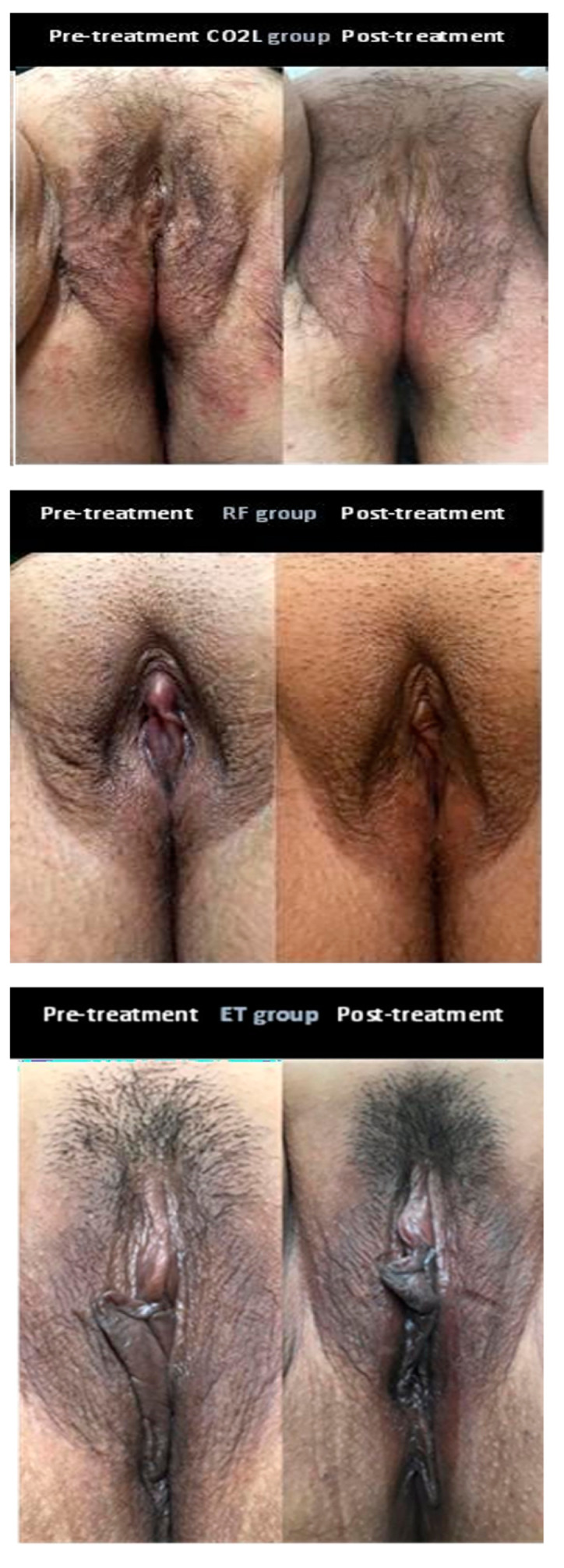
Participants’ vulva pictures before and after treatment per group.

Regarding clinical aspects of the vulva, a significant change was observed (*p* = 0.017) after all treatments (*p* = 0.918), with a reduction in the atrophic global vulvar aspect and an enhancement of the trophic aspect. This was confirmed by a photographic evaluation which also revealed improvement in vulvar rugae and tropism, skin color and reduction in erythema (Table 2 and Figure 2). Moreover, high satisfaction was also reported after treatment according to the Likert scale evaluation: CO_2_L (4.55 ± 0.97), RF (4.54 ± 0.95), CT (4 ± 1.41), *p* = 0.066. The reported satisfaction rate was 15/20 (75%) in the CO_2_L group, 15/17 (93.8%) in the RF group and 7/11 (63.6%) in the ET group.

Histological evaluation revealed enhanced dermal papillae before pre-treatment, significantly reducing post-treatment in all groups (*p* = 0.002). Furthermore, it was also identified that hyperkeratosis reduced post-treatment in the CO_2_L and RF groups and not in the ET group (*p* = 0.02), but in the ET group, the mean epithelial thickness increased in all three groups (*p* < 0.001) (Table 3). Moreover, upon stromal evaluation, the vascular number and distribution showed normal patterns before and after treatment (Table 3). Pain-motivated treatment discontinued in five women: 2/32 (6.25%) in the CO_2_L group and 3/24 (12.5%) in the RF group. Vulvar pain decreased during the treatment sessions (*p* = 0.012) and was lower in the CO_2_L group than in the RF group (*p* < 0.001), independent of the session (Table 4). No unintended effects were reported by the participants or observed during gynecological examinations during treatment and follow-up visits. Melanosis was not identified histologically or clinically after any treatment. None of the patients had complications related to energy use, such as burns, damage to organs adjacent to the vagina, or skin hypo- or hyperpigmentation. Despite the report of herpes in three women who received antiviral prophylaxis, none had re-activation of the infection during the study.

## 4. Discussion

This is the first study involving the use of CO_2_L, RF and ET for the treatment of GSM to evaluate clinical and histological aspects of the vulvar vestibule. This region is related to GSM signs and symptoms such as pruritus, fissure, reduced turgor and tropism and dyspareunia. Our findings demonstrated that the CO_2_L and RF groups showed a similar result to estrogen therapy, the current gold standard treatment [14], in terms of clinical and histological evaluation criteria.

The women included in this study presented moderate-to-severe GSM. We identified a reduction in the intensity of these complaints and their absence after treatment. These findings are consistent with the gynecological examination and photographic evaluation made by specialists blinded to group allocation and evaluation. This confirms the agreement between the patients’ and professionals’ impressions, and corroborates the data previously verified by other authors who demonstrated a significant improvement in GSM with energy treatments [9,15,16,17].

The women in this study were predominantly sexually active. This could have been the main motivation for seeking treatment since the mean VAS score for dyspareunia was the highest parameter evaluated pre-treatment. The impact of those treatments on women’s sexual lives is notorious, with dyspareunia showing the greatest post-treatment improvement.

GSM improvement was achieved in all groups and confirmed by high post-treatment satisfaction. This corroborates other studies in which participants reported being satisfied or very satisfied with CO_2_L and RF treatments [7,17,18].

Women with GSM are expected to present histological atrophy, defined as a reduction in the number of epithelial layers, epithelial thickness, cell maturation, dermal papillae number and stromal vascularity [19]. All women included in this study were postmenopausal (5.54 mean years) and had moderate-to-severe GSM symptoms. However, the histologic atrophy criteria described above were not identified in the vestibule vulvar biopsies of pre-treated women, evidencing a discrepancy between clinical and histological findings.

Our histological analysis of 48 women showed an increase in the number of pre-treatment dermal papillae in more than half of the sample (31/48—64.6%), and a significant post-treatment reduction in this finding toward normalization. This suggests that these signs may be related to the presence of symptoms. Hyperkeratosis was also observed in the same proportion as before treatment (30/48—62.7%). This sign is related to reduced skin elasticity and may be associated with fissuring, pruritus and dyspareunia.

After treatment, an increase in epithelial thickness was observed, corroborating the effect already demonstrated by other authors who evaluated the histological effect of CO_2_L, albeit on the vaginal mucosa [20,21] rather than keratinized epithelium of the vulvar vestibule.

Concerning stromal evaluation, the number of capillaries was normal or increased rather than reduced. Further, it presented a predominantly homogenous distribution pre-treatment which was maintained post-treatment. These findings suggest that histological atrophy may occur later and may not be associated with GSM symptoms.

On the other hand, the epithelial or stromal cells showed no structural damage such as fibrosis, melanosis or layer reduction after energies were applied to the vestibule vulvar. Following clinical findings, no major complications were reported previously [7,8].

Pain during treatments may reduce adherence. Topical local anesthetics (4% lidocaine) were applied to the vulvar vestibule region to ensure safety and a low risk of intoxication. However, pain was the reason for treatment discontinuation in 6.5% of patients in the CO_2_L group and 12.5% of patients in the RF group. The pain was considered moderate during the first CO_2_L session, with a reduction to mildness during the subsequent sessions. In the RF group, pain was moderate-to-severe in the first session and reduced to moderate in the subsequent sessions, suggesting that CO_2_L was better tolerated than RF regarding pain.

A limitation of this study was the collection of biopsy specimens using the Gayllor-Medina forceps, which were not ideal for obtaining high-quality samples, resulting in a related sample loss of 11% (i.e., eight cases with inadequate specimens for analysis). Hematoxylin and eosin staining was used exclusively for hystomorphometric analysis. This is not the most appropriate for the stromal component evaluation (such as collagen, elastin, and glycosaminoglycans) or vascularization. However, it does allow for an initial structural evaluation. Supporting this, some already published studies investigated the same histological parameters as our study, utilizing the same staining agent (hematoxylin and eosin) [22,23].

Moreover, these histological findings may be relevant once they were evaluated in 48 symptomatic postmenopausal women, while most studies to date have described tissue effects using animal models or cohorts with smaller numbers of women [17,24].

This work was mostly conducted during the COVID-19 pandemic, making participant recruitment, treatment and follow-up difficult, as participants were required to attend five presential visits during the first phase of the study. Although GSM impairs the quality of life, it is not life-threatening and this has delayed recruitment and impaired adherence. In our case, this resulted in 13.7% of the cohort being lost. In addition, access to outpatient clinics and laboratories was restricted during this period, making it difficult to process and analyze the samples.

One of the main challenges in studying GSM treatment is to evaluate lasting positive results. Although there is still no consensus, it has been reported that the duration of the effect of CO_2_L on GSM is 12 months [25]. In a comparative study of erbium laser and topical estriol, Gaspar et al. (2017) reported that the positive effect of erbium laser remained significant after 12 to 18 months, while the treatment effects with estradiol decreased in the same period [26]. In the present study, the effects were evaluated for only 120 days. Thus, the lasting positive effects and the need for reapplications are still uncertain and require further evaluation.

Strategies to treat GSM, such as CO_2_L and RF, achieve clinical outcomes similar to hormonal treatment, which is the gold standard. However, the histological findings with an anatomic–clinical dissociation showed tissue modifications pre- and post-treatment, suggesting the need to pursue further investigation with immunohistochemical and molecular analyses to better understand the mechanisms of action of the energies as the functional aspects of GSM.

Furthermore, recent research [22,27,28] also demonstrated that CO2 laser as well as RF therapy leads to a significant reduction in clinical symptoms related to GSM in postmenopausal cancer survivors.

Indeed, in breast cancer survivors, atrophic vaginitis/vaginal dryness can affect up to 70% of postmenopausal patients leading to the genitourinary syndrome of menopause (GSM). After surgery, these patients receive additional chemo, hormonal and radiation therapy, which can aggravate vaginal symptoms. In this case, these therapies can represent a valuable non-hormonal therapeutic approach in the management of GSM.

Particularly, already published studies reported that the use of vaginal fractional CO_2_ laser is a cost-effective strategy for the treatment of symptoms such as dyspareunia, associated with GSM. [29]. In the model reported in this study, the vaginal CO_2_ laser was the optimal and cost-effective strategy, and it was proposed to consider the possibility of providing insurance coverage for this treatment option. Practical implications and future research direction will focus on the safe use of vaginal laser and RF treatments for patients suffering from hyperkeratosis associated with the condition of GSM or patients who cannot support hormone therapy, thus encouraging hospital systems, private insurance companies and state insurers to consider covering vaginal energy-based device treatments for patients, as required by the FDA.

### Study Limitation

In this research, the sample size determination was linked to the histological investigations which represent a predominant part of this research, since a limited number of patients can be subjected to biopsy.

## 5. Conclusions

In conclusion, CO_2_L, RF and ET significantly improved GSM concerning the vulvar vestibule at the 4 months follow-up. Post-treatment histological modifications were similarly observed in all groups, except for hyperkeratosis reduction observed only in the energy-treated groups. No tissue damage was identified upon clinical or histological evaluations.

## Figures and Tables

**Table 1:** Clinical and demographic pre-treatment parameters.

	Group			*p*
	CO_2_L (*N* = 20)	BF (*N* = 17)	ET (*N* = 11)	
Age (years)				
mean ± SD	54.9 ± 4.8	54.2 ± 6.2	54.6 ± 3.9	0.937 *
Menopause period (years)				
mean ± SD	5.1 ± 3.2	4.7 ± 2.7	4.9 ± 3.1	0.953 £
Parity				
mean ± SD	1.6 ± 0.9	2 ± 1.1	2.5 ± 1.3	0.144 £
BMI (Kg/m²)				
mean ± SD	28.5 ± 6.4	28.2 ± 2.7	28.8 ± 3	0.963 *
Ethnicity				
White	13 (65%)	9 (52.9%)	10 (90.9%)	0.082 #
Not White	7 (35%)	8 (47.1%)	1 (9.1%)	
Sexually active	18 (90%)	17 (100%)	11(100%)	0.828 #

Mean (±standard deviation); BMI, body mass index; * t-Student; £ Mann–Whitney; # Likelihood ratio test.

**Table 2:** Clinical evaluation based on the patients’ photos and gynecologic exam.

	Group			*p*_Group_	*p*_time_	*p*_group×time_
	CO_2_L (*N* = 20)	RF (*N* = 17)	ET (*N* = 11)			
Trofism/vulvar rugae				0.839	0.010	0.349
Pre	2.9 ± 0.9	3.3 ± 1.1	3.2 ± 0.9			
Post	3.6 ± 0.5	3.5 ± 0.6	3.5 ± 1.2			
Fissure				0.064	0.240	0.932
Pre	4.7 ± 0.7	4.8 ± 0.3	4.9 ± 0.2			
Post	4.7 ± 0.5	4.9 ± 0.1	5 ± 0.1			
Erythema				0.059	0.037	0.779
Pre	3.7 ± 0.8	4.2 ± 0.9	3.5 ± 0.7			
Post	4 ± 0.8	4.3 ± 0.6	3.8 ± 0.4			
Vulva’s skin color				0.811	<0.001	0.796
Pre	3 ± 0.8	3.1 ± 1.2	3.3 ± 0.7			
Post	3.6 ± 0.4	3.6 ± 0.5	3.6 ± 0.6			
Vulva clinical aspect				0.918	0.017	0.186
Pre *n* (%)						
Trophic	3 (15)	10 (58.8)	4 (36.4)			
Hypotrophic	11 (55)	3 (17.6)	6 (54.5)			
Atrophic	6 (30)	4 (23.5)	1 (9.1)			
Post *n* (%)						
Trophic	15 (75)	9 (52.9)	8 (72.7)			
Hypotrophic	5 (25)	7 (41.2)	2 (18.2)			
Atrophic	0 (0)	1 (5.9)	1 (9.1)			

Values expressed by the mean and standard deviation (SD); generalized estimating equations (GEE).

**Table 3:** Pre- and post-treatment histological parameters.

	Group			*p*_Group_	*p*_Time_	*p*_Group×time_
	CO_2_L (*N* = 20)	RF (*N* = 17)	ET (*N* = 11)			
Dermal papillae (*n*/%)				0.426	0.002	0.591
Pre	12 (60)	13 (76.5)	6 (54.5)			
Post	9 (45)	8 (47.1)	3 (27.3)			
Hyperkeratosis (*n*/%)				0.001	0.020	0.244
Pre	17 (85)	5 (29.4)	8 (72.7)			
Post	11 (55)	1 (5.9)	8 (72.7)			
Medium thickness (mm)				0.514	<0.001	0.619
Pre (mean ± SD)	2.3 ± 0.7	2.2 ± 0.6	2.1 ± 0.6			
Post (mean ± SD)	2.9 ± 1	2.6 ± 0.8	2.7 ± 0.7			
Number of vessels (*n*/%)				0.882	0.807	0.772
Pre						
Enhanced	3 (15)	3 (17.6)	2 (18.2)			
Normal	17 (85)	14 (82.4)	9 (81.8)			
Post						
Enhanced	4 (20)	3 (17.6)	1 (9.1)			
Normal	16 (80)	14 (82.4)	10 (90.9)			
Vascular distribution (*n*/%)				&	&	&
Pre						
Subepithelial	1 (5)	2 (11.8)	0 (0)			
Uniform	19 (95)	15 (88.2)	11 (100)			
Post						
Subepithelial	3 (15)	1 (5.9)	1 (9.1)			
Uniform	17 (85)	16 (94.1)	10 (90.9)			

Values expressed by the mean and standard deviation (SD); generalized estimating equations (GEE); & cannot be estimated by the presence of very low frequencies of more than one variable.

**Table 4:** Vulvar pain during the energy application sessions.

	Group		*p*_Group_	*p*_Time_	*p*_Group×time_
	CO_2_L (*N* = 20)	RF (*N* = 17)			
Vulvar pain			<0.001	0.012	0.720
1st session (mean ± SD)	4.7 ± 2.4	6.8 ± 2.3			
2sd session (mean ± SD)	2.9 ± 2.6	5.8 ± 3.3			
3rd session (mean ± SD)	3.1 ± 2.4	5.4 ± 2.8			

Values expressed by the mean and standard deviation (SD); generalized estimating equations (GEE).

## Data Availability

Data that support the study findings are available on request from the corresponding author.
